# Construction and validation of a prediction model for in-stent restenosis following coronary stent implantation during dual antiplatelet therapy

**DOI:** 10.3389/fcvm.2026.1780572

**Published:** 2026-05-04

**Authors:** Zhuoyi Zhang, Yalin Huang, Yumin Ye, Zhenhua Wang

**Affiliations:** Department of Cardiology, The Second Affiliated Hospital of Fujian Medical University, Quanzhou, Fujian, China

**Keywords:** antiplatelet therapy, coronary heart disease (CHD), in-stent restenosis (ISR), nomogram, percutaneous coronary intervention (PCI), thromboelastography (TEG)

## Abstract

**Objectives:**

In-stent restenosis (ISR) is a serious complication that occurs after a percutaneous coronary intervention (PCI). This study aims to integrate thromboelastography (TEG) parameters, coagulation function, and clinical indicators to develop and validate a prediction model for ISR during antiplatelet therapy after PCI.

**Method:**

A total of 401 consecutive patients with coronary artery disease who underwent a PCI with drug-eluting stent implantation between January 2018 and January 2024 were enrolled in this study. Clinical baseline characteristics were collected. The Least Absolute Shrinkage and Selection Operator (LASSO) regression was used to screen 6 key predictors from 34 candidate clinical features. Multivariate logistic regression identified independent risk factors for ISR. Model performance was evaluated using the receiver operating characteristic curve for discrimination, bootstrap internal validation for stability, the Hosmer–Lemeshow test for goodness of fit, and decision curve analysis for clinical utility.

**Results:**

Among the 401 enrolled patients, 137 (34.2%) developed ISR. LASSO regression selected 6 key predictors from 34 candidate variables. Multivariable logistic regression ultimately identified a low adenosine diphosphate (ADP) inhibition rate, a low arachidonic acid (AA) inhibition rate, reaction time (*R*-value), and age as independent risk factors for ISR. The constructed nomogram demonstrated good discrimination (area under the curve = 0.752, 95% confidence interval: 0.702–0.803), stable performance upon bootstrap internal validation, and satisfactory calibration (Hosmer–Lemeshow test, *P* = 0.320). Furthermore, decision curve analysis indicated a favorable clinical net benefit across a broad range of threshold probabilities.

**Conclusions:**

The prediction model, constructed based on TEG parameters (ADP inhibition rate, AA inhibition rate, *R*-value), age, and N-terminal pro-B-type natriuretic peptide, effectively identifies patients at high risk for ISR after a PCI. It provides a basis for individualized antiplatelet therapy and postoperative management decisions.

## 1 Introduction

Coronary artery disease (CAD) remains a leading global cause of cardiovascular mortality (1, 2), making vascular reconstruction techniques a focal area in coronary heart disease (CHD) research. Percutaneous coronary intervention (PCI), with its minimally invasive nature and ability to achieve rapid revascularization (3, 4), is not only the primary treatment for acute coronary syndrome (5) but also facilitates a multitechnique synergy under precise imaging guidance (6), playing an indispensable role in improving the quality of life and long-term prognosis of patients. Over the past 40 years, with the maturation and development of PCI technology, bare-metal stents (BMS) overcame the issue of late vessel occlusion seen with traditional balloon angioplasty (BA), significantly reducing post-PCI event rates (7). However, vascular injury induced by BMS implantation and the progression of coronary atherosclerosis can lead to excessive neointimal hyperplasia (8–10), with in-stent restenosis (ISR) rates reaching up to 40% (11). The advent and continuous optimization of drug-eluting stents (DES) have reduced ISR rates to 3%–20% (12, 13).

Risk factors for ISR after a PCI can be systematically categorized into preprocedural and postprocedural factors. Preprocedural factors include underlying conditions (diabetes, hypertension), advanced age, long-term smoking, morphological characteristics of the target vessel, lesion location, stenosis severity, and abnormalities in certain blood biochemical markers related to inflammation and lipid metabolism (14–17). Postprocedural factors include non-standardized antiplatelet therapy (18) and antiplatelet drug resistance or low response (19). Regular and effective antiplatelet therapy is crucial after a PCI. Predictive models offer the advantage of enabling more precise risk assessment through the standardized integration of risk factors. Therefore, establishing a predictive model for ISR after DES implantation in patients with CAD is essential.

Although previous studies have analyzed potential predictors of ISR and developed ISR-related nomograms for patients with PCI, existing models have limitations. First, most models focus on the impact of preprocedural factors on ISR occurrence, ignoring the importance of effective postprocedural antiplatelet therapy and thus missing optimal intervention timing (17, 20, 21). Second, current models incorporating antiplatelet drug response rates lack dynamic monitoring of coagulation parameters during treatment (17) and do not validate drug responsiveness, posing a risk of false positives. Finally, most models lack clearly defined follow-up periods for patients developing ISR. Currently, prediction models based on postprocedural platelet function and coagulation-related indicators remain scarce. Against this background, this study aims to analyze demographic characteristics, clinical data, laboratory parameters, lipid profiles, coagulation function, and thromboelastography (TEG) indicators in patients with CAD 12 months post-PCI. The findings will provide new evidence for constructing a postprocedural risk factor nomogram, aiding clinicians in identifying high-risk ISR patients and optimizing treatment strategies.

## 2 Materials and methods

### 2.1 Study population

**Figure 1:**
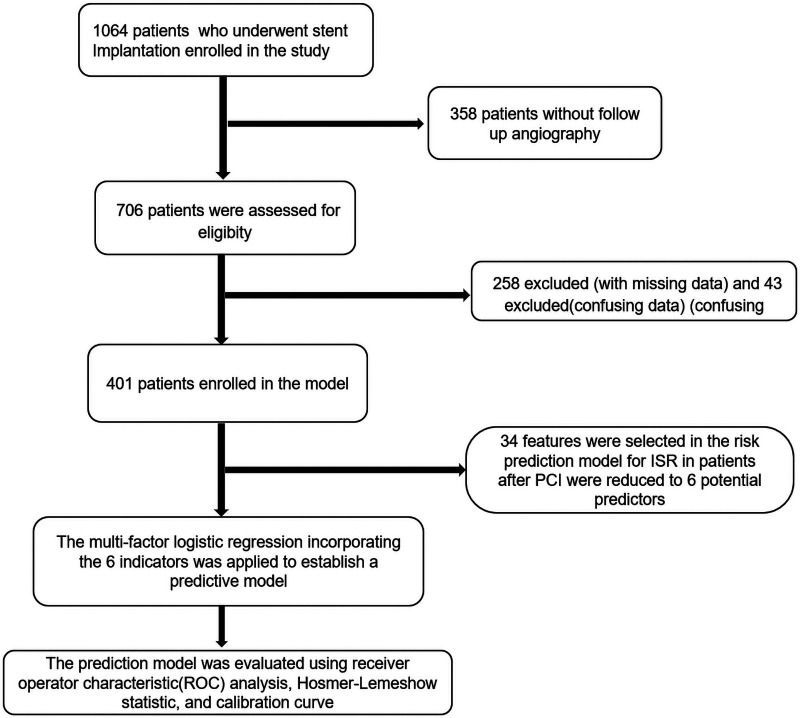
The study design and the selection procession of patients with CAD.

This retrospective cohort study was approved by the Ethics Committee of the Second Affiliated Hospital of Fujian Medical University (Ethics Approval No.: 2025-358) and conducted in accordance with the principles of the Declaration of Helsinki. Patient informed consent was waived. From 1,064 consecutive patients who underwent coronary stent implantation at our hospital between January 2018 and January 2024, 401 patients with CAD who met the inclusion and exclusion criteria were finally enrolled (Figure 1).

#### 2.1.1 Inclusion criteria

The inclusion criteria were patients who regularly received ≥12 months of dual antiplatelet therapy (DAPT, aspirin combined with a P2Y12 receptor antagonist) and statin therapy and completed coronary angiography follow-up within 12–14 months postprocedure.

#### 2.1.2 Exclusion criteria

The exclusion criteria were patients with a history of coronary artery bypass grafting (CABG); NYHA class IV heart failure or acute decompensated heart failure; active infection (e.g., pneumonia and gastroenteritis); hepatic failure (Child–Pugh class C); autoimmune diseases (e.g., systemic lupus erythematosus and Sjögren's syndrome); hematological disorders (e.g., leukemia, lymphoma, and coagulation dysfunction); malignancy or life expectancy <1 year; severe arrhythmia requiring long-term anticoagulation; pharmacogenetic testing results inconsistent with TEG efficacy results; laboratory data during hospitalization for acute myocardial infarction; missing key data or loss to follow-up.

### 2.2 Clinical data collection

Employing a case–control design, the primary endpoint was ISR occurrence, defined as ≥50% diameter stenosis within the stent or at its edges (within 5 mm) due to neointimal hyperplasia, thrombosis, or vascular remodeling (22). All patients underwent a coronary angiography within 12–14 months after a PCI. Collected clinical data included: (1) Demographics: age, gender, smoking history; (2) Clinical Data: hypertension, diabetes, type of antiplatelet medication; (3) Laboratory Parameters: platelet count (PLT), lipid profile [total cholesterol (TC), triglycerides (TG), high-density lipoprotein cholesterol (HDL-C), low-density lipoprotein cholesterol (LDL-C)], renal function (creatinine, Cr), liver function [total bilirubin (TBil), alkaline phosphatase (ALP)], coagulation function [prothrombin time activity (PTA), fibrinogen (FIB), D-Dimer, thrombin time (TT)], TEG parameters [reaction time (*R*-value), maximum amplitude (MA), coagulation index (CI), adenosine diphosphate (ADP)/arachidonic acid (AA) inhibition rates], pharmacogenetic test results; (4) Coronary Angiography: restenosis lesion characteristics.

All TEG evaluations were conducted at least 6 months following a PCI while patients were on a stable DAPT regimen. This timing was deliberately chosen to accurately reflect platelet function and coagulation status during steady-state maintenance therapy. Venous blood was drawn into standardized sodium citrate vacuum tubes. To minimize technical variability, all samples were processed within 2 h of collection by uniformly trained laboratory personnel strictly adhering to standard operating procedures. The TEG parameters evaluated in this study were selected based on their mechanistic links to the pathophysiology of ISR. The ADP and AA inhibition rates directly quantify the efficacy of P2Y12 receptor antagonists and aspirin in suppressing platelet aggregation. A reduction in these rates indicates functional antiplatelet “hyporesponsiveness,” a state that perpetuates platelet activation and subsequent local inflammation and neointimal hyperplasia. In addition, the *R*-value, defined as the latency from assay initiation to initial fibrin clot formation, provides a comprehensive measure of coagulation factor activity. A shortened *R*-value is indicative of a hypercoagulable state post-PCI, which promotes restenosis through the activation of both platelets and the vascular endothelium. To minimize false positives, a rigorous composite definition was applied: a “low ADP inhibition rate” required an observed ADP inhibition of <20% combined with the carriage of at least one CYP2C19 loss-of-function allele (e.g., *2 or *3). Likewise, a “low AA inhibition rate” was defined as an AA inhibition of <30% paired with homozygous mutations in either the PEAR1 or the GP1BA gene.

### 2.3 Statistical analysis

Clinical data were analyzed using R software (version 4.5.0) and SPSS (version 25.0). Normally distributed continuous variables were expressed as mean ± standard deviation (SD) and compared using independent samples t-tests. Non-normally distributed continuous variables were expressed as median (interquartile range, IQR) and compared using non-parametric tests. Categorical variables were expressed as frequency (percentage) and compared using chi-square tests. Statistical significance was set at *α* = 0.05 (*P* < 0.05). Least Absolute Shrinkage and Selection Operator (LASSO) regression (using the glmnet package in R) with 10-fold cross-validation screened 6 ISR predictors from 34 candidate variables. Multivariate logistic regression identified independent risk factors for ISR; variance inflation factors (VIFs) were calculated to exclude multicollinearity. The final clinical prediction model was constructed based on the relative weights of the risk factors, visualized as a nomogram. The model's discriminatory ability was assessed using the receiver operating characteristic (ROC) curve and area under the curve (AUC). Calibration curves were plotted. Internal validation was performed using the Bootstrap resampling method (B = 1,000 repetitions). Clinical effectiveness was evaluated using decision curve analysis (DCA).

## 3 Results

### 3.1 Baseline clinical characteristics analysis

Based on inclusion/exclusion criteria, 401 patients with CAD with complete follow-up data were selected from a total of 1,064 patients, with the breakup being 137 ISR patients and 264 non-ISR patients. Baseline characteristics are given in Table 1. No significant differences were found between groups regarding gender, smoking history, hypertension, diabetes, TBil, ALP, fasting blood glucose (FBG), Cr, TC, TG, HDL, LDL, apolipoprotein A (APOa), apolipoprotein B (APOb), PLT, and some coagulation parameters [clot formation time (*K*-value), α-angle, MA, global coagulation index (*G*-value), estimated percent lysis (EPL), percent lysis at 30 min (Ly30), PTA, PTA ratio, activated partial thromboplastin time (APTT), APTT ratio, TT] (all *P* > 0.05). Significant differences were observed in terms of age, N-terminal pro-B-type natriuretic peptide (NT-proBNP), TEG *R*-value, TEG CI, FIB, and D-dimer (all *P* < 0.05).

**Table 1:** The baseline characteristics of the patients.

Variables	No-ISR (*n* = 264)	ISR (*n* = 137)	Statistic	*P*
Age	61.83 ± 10.23	64.83 ± 10.88	*t* = −2.73	0.007
Male	219 (82.95)	111 (81.02)	*χ*^2^ = 0.23	0.631
Smoking	103 (39.16)	55 (40.15)	*χ*^2^ = 0.04	0.849
Hypertension	162 (61.36)	79 (57.66)	*χ*^2^ = 0.51	0.473
Diabetes	96 (36.36)	57 (41.61)	*χ*^2^ = 1.05	0.305
Low ADP inhibition rate	114 (43.35)	98 (71.53)	*χ*^2^ = 28.73	<0.001
Low AA inhibition rate	16 (6.08)	41 (30.15)	*χ*^2^ = 42.39	<0.001
TBil, μmol/L	10.32 (8.11–13.23)	10.08 (7.68–12.32)	Z = −1.43	0.153
ALP, U/L	68.70 (58.30–84.05)	67.00 (57.90–84.50)	Z = −0.04	0.971
FBG, mmol/L	5.52 (4.98–6.43)	5.69 (4.98–6.81)	Z = −0.95	0.342
Cr, μmol/L	82.00 (71.00–95.42)	85.80 (68.00–98.00)	Z = −0.59	0.552
TC, mmol/L	3.80 (3.19–4.65)	4.07 (3.17–4.90)	Z = −1.02	0.308
TG, mmol/L	1.31 (0.94–2.07)	1.29 (0.97–1.82)	Z = −0.27	0.791
HDL, mmol/L	0.96 (0.80–1.17)	0.91 (0.80–1.18)	Z = −0.35	0.729
LDL, mmol/L	2.15 (1.62–2.79)	2.23 (1.67–2.95)	Z = −0.60	0.546
APOa, g/L	1.13 (0.97–1.30)	1.13 (0.97–1.26)	Z = −0.75	0.454
APOb, g/L	0.81 (0.64–1.04)	0.89 (0.68–1.08)	Z = −1.39	0.164
NT-proBNP, pg/mL	54.83 (40.53–160.75)	120.00 (50.00–445.00)	Z = −3.61	<0.001
PLT, 10^9^/L	220.00 (186.75–257.00)	213.00 (186.00–260.00)	Z = −0.63	0.532
*R*-value, min	5.82 ± 1.11	5.39 ± 1.19	t = 3.56	<0.001
*K*-value, min	1.80 (1.40–2.10)	1.70 (1.30–2.20)	Z = −0.94	0.347
α-Angle, °	65.40 (60.63–69.23)	66.20 (60.70–69.60)	Z = −0.65	0.517
MA, mm	60.70 (55.60–64.60)	60.30 (54.80–65.30)	Z = −0.10	0.924
*G,* d/sc^a^	7,764.30 (6,461.35–9,174.17)	7,688.30 (6,202.50,9,500.00)	Z = −0.02	0.987
EPL, %	0.000 (0.000–0.000)	0.000 (0.000–0.000)	Z = −1.39	0.164
Ly30%	0.000 (0.000–0.000)	0.000 (0.000–0.000)	Z = −1.29	0.198
CI	0.20 (0.00–1.30)	1.30 (0.00–2.50)	Z = −4.35	<0.001
PTA, %	101.10 (93.60–108.60)	100.50 (93.30–108.60)	Z = −0.35	0.723
PTA ratio	0.99 (0.94–1.04)	0.99 (0.94–1.03)	Z = −0.25	0.801
APTT, s	27.50 (24.90–30.20)	27.50 (25.30–30.50)	Z = −0.66	0.510
APTT ratio	0.95 (0.85–1.04)	0.96 (0.85–1.08)	Z = −0.64	0.523
FIB, g/L	2.81 (2.44–3.37)	3.01 (2.53–3.83)	Z = −2.78	0.005
D-Dimer, μg/mL	0.25 (0.15–0.50)	0.34 (0.24–0.88)	Z = −3.97	<0.001
TT, s	17.20 (16.30–18.00)	17.10 (15.90–18.30)	Z = −0.20	0.841

Distribution of continuous variables was assessed using the Shapiro–Wilk test. Normally distributed variables are presented as mean ± standard deviation (SD). Non-normally distributed variables are presented as median (IQR). For the two normally distributed variables with unequal variances between groups (age and *R*-value), Welch's t-test was used for between-group comparisons.

^a^ The G' value represents the elastic modulus strength of the blood clot under low-amplitude oscillatory shear stress. The unit d/sc stands for dynes per square centimeter (dynes/cm²), which is employed to quantify the mechanical strength of the blood clot in resisting deformation.

### 3.2 Variable screening

**Figure 2:**
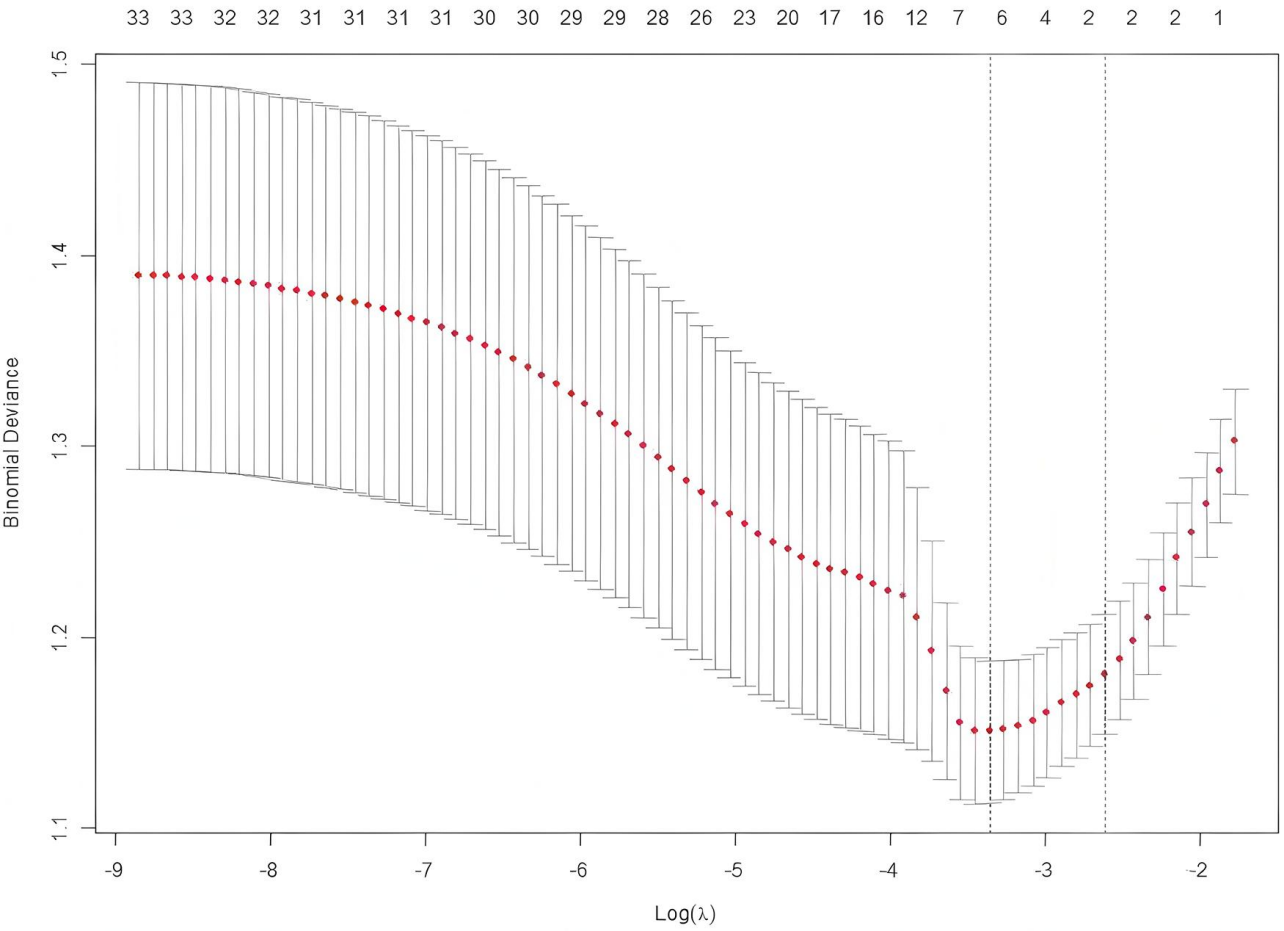
Risk factors selected using the LASSO model. Optimal parameter (lambda) selection for the LASSO model was cross validated using the minimum criterion. Partial likelihood deviation (binomial deviation) curves vs. log (lambda). Dotted vertical lines are drawn at the best values of 1SE (1-SE criterion) using the minimum criterion and the maximum criterion.

**Figure 3:**
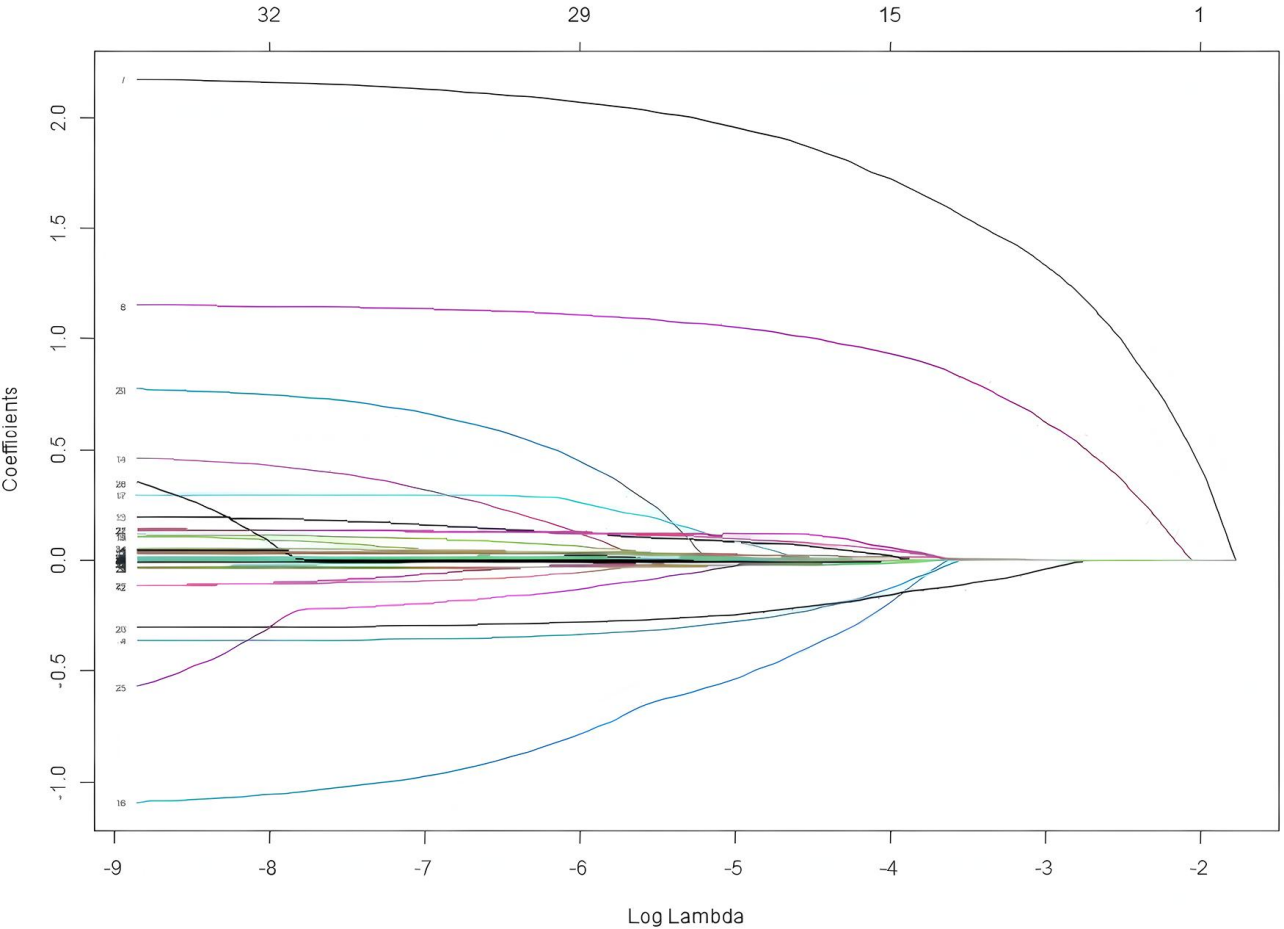
LASSO coefficient profiles for the 34 candidate clinical features. As the regularization parameter increases, the coefficients are progressively shrunk toward zero. At the optimal lambda value selected via 10-fold cross-validation, exactly six predictors retain non-zero coefficients, while the others are eliminated. This penalization process effectively simplifies the model and ensures the identification of the most robust clinical predictors.

Using LASSO regression analysis, we screened 6 non-zero coefficient predictors from the 34 initial features: decreased ADP inhibition rate, decreased AA inhibition rate, age, NT-proBNP, TEG *R*-value, and TEG CI (Figures 2, 3).

### 3.3 Risk factor analysis and prediction model construction

**Figure 4:**
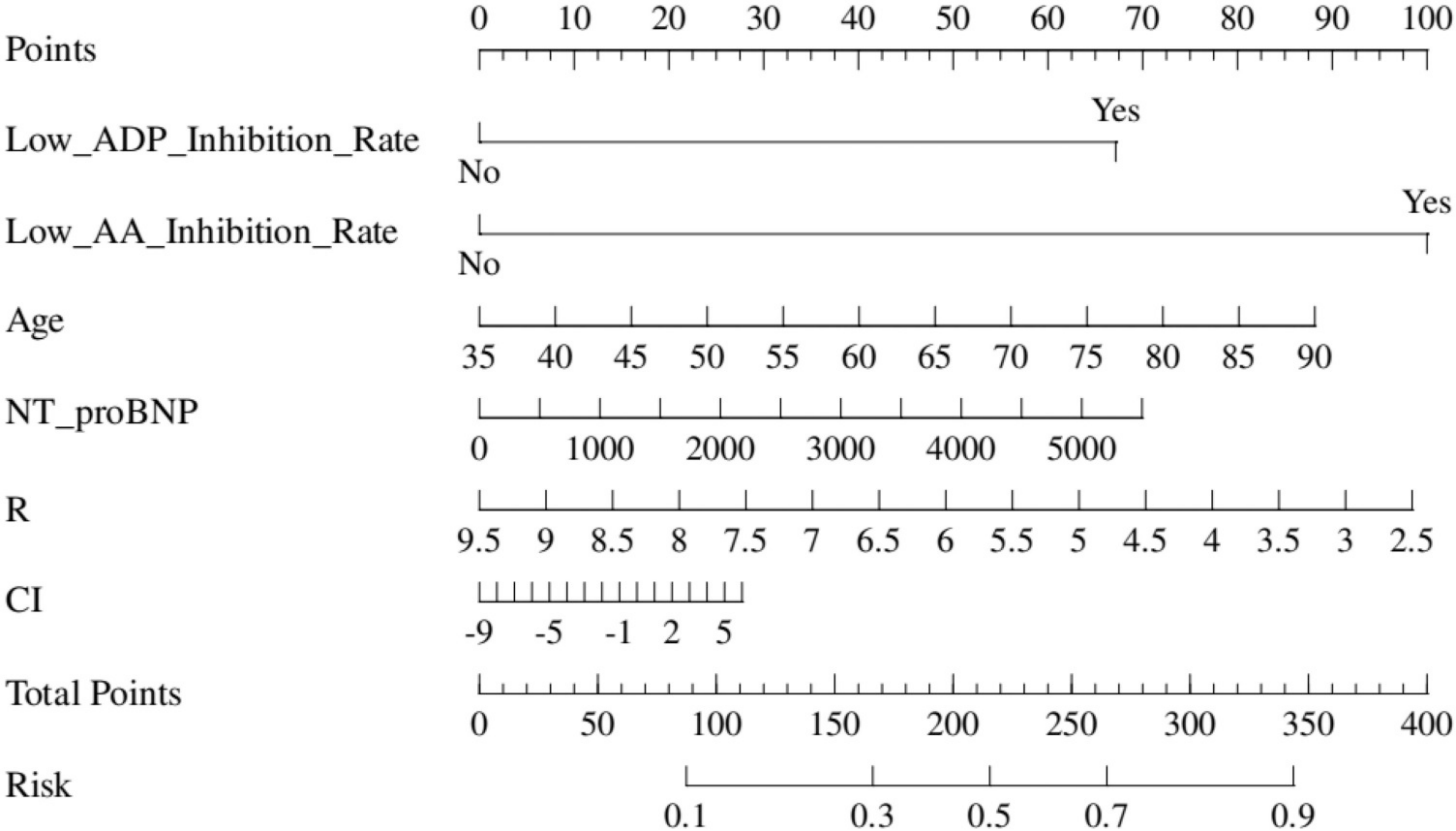
A nomogram to predict the probability of ISR in patients with stent implantation after a PCI. The nomogram included the factors of lower ADP inhibition rate, lower AA inhibition rate, age, the reaction time of thromboelastograph, N-terminal pro-brain natriuretic peptide, and the coagulation index of thromboelastography.

Using ISR occurrence as the dependent variable and the six LASSO-selected predictors as independent variables, we performed logistic regression. VIF values showed no significant multicollinearity. The final prediction model equation was

$$\begin{array}{rl} logit(P) & =-2.08+1.15\times (X\_ADP)+1.72\times (X\_AA)+0.03\\ & \;\times (X\_Age)-0.24\times (X\_R)+0.0002\\ & \;\times (X\_NT\text{-}proBNP)+0.03\times (X\_CI) \end{array}$$

where *X_ADP =* 1 if ADP inhibition rate is low, otherwise, 0; *X_AA* *=* 1 if the AA inhibition rate is low, otherwise, 0; *X_Age, X_NT-proBNP, X_R, and X_CI* represent the actual observed values of age (years), NT-proBNP (pg/mL), *R*-value (min), and coagulation index, respectively. The nomogram is shown in Figure 4. Logistic regression results identified decreased ADP inhibition rate, decreased AA inhibition rate, advanced age, and shortened TEG *R*-value as independent risk factors for ISR. TEG CI and NT-proBNP were not independent risk factors (Table 2).

**Table 2:** Logistic regression analysis of the predictors of ISR.

Characteristics	*P*	OR	95% confidence interval	VIF
Low ADP inhibition rate	<0.001	3.16	1.95–5.14	1.049
Low AA inhibition rate	<0.001	5.56	2.85–10.80	1.055
Age	0.017	1.03	1.01–1.05	1.040
*R*-value	0.029	0.79	0.63–0.98	1.126
NT-proBNP	0.118	1.00	1.00–1.00	1.048
CI	0.639	1.03	0.90–1.18	1.200

### 3.4 Model validation and evaluation

**Figure 5:**
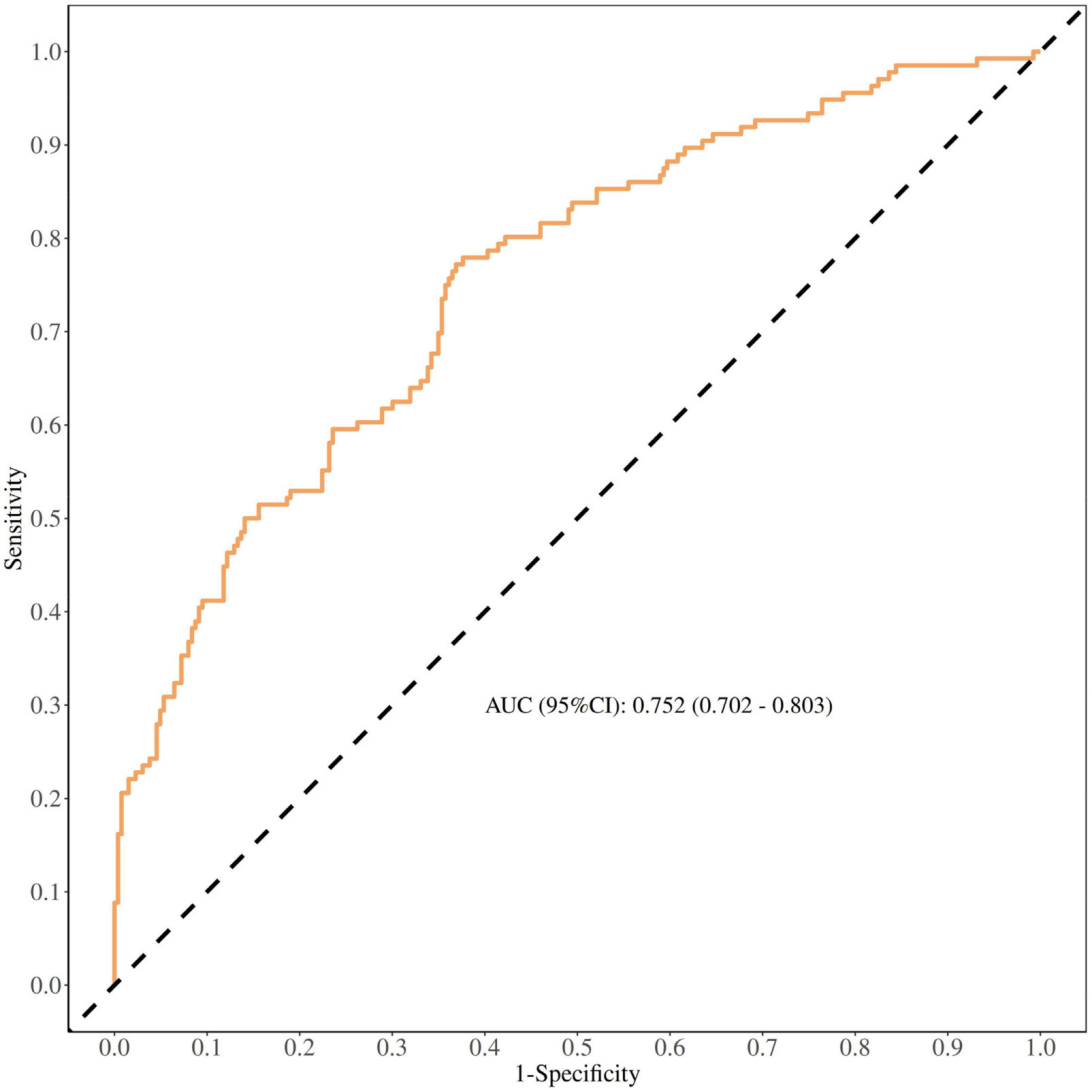
ROC curves for validating the discrimination power of a nomogram.

**Figure 6:**
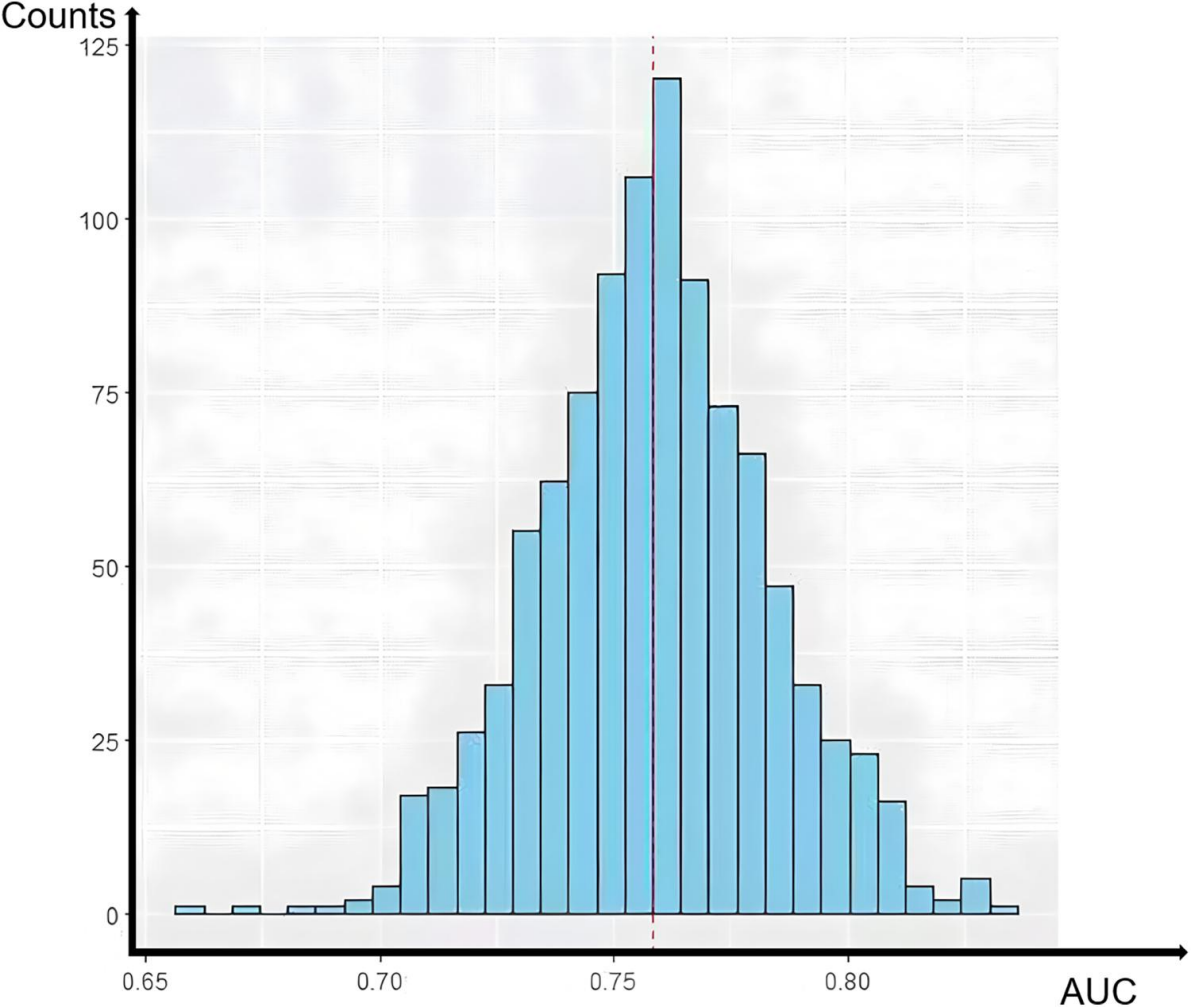
A histogram of the area under the receiver operating characteristic curve (AUC) values derived from 1,000 bootstrap internal validation replicates of the ISR prediction nomogram. This histogram displays the distribution of AUC values across 1,000 bootstrap resamples performed for internal validation. The *x*-axis represents the AUC value obtained in each bootstrap replicate; the *y*-axis indicates the frequency (number of replicates). The distribution approximates a normal distribution with a peak (mode) at approximately 0.75 and a mean of 0.758 (95% confidence interval 0.709–0.807). Importantly, 94.6% of the bootstrap AUC values fell within the narrow range of 0.70–0.80, demonstrating excellent stability and robustness of the model's discriminative performance.

**Figure 7:**
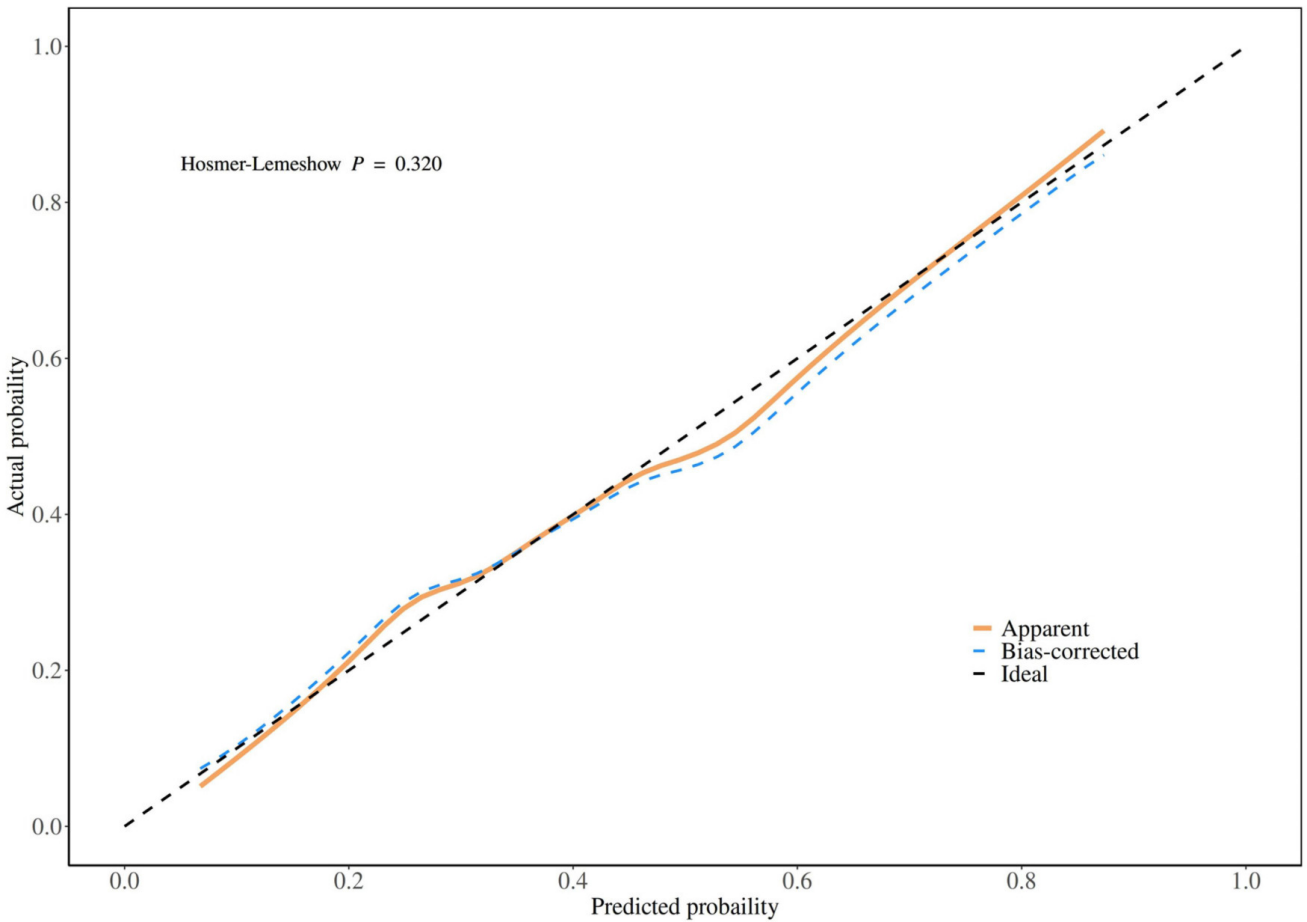
Calibration plots of the nomogram for the probability of PCI patients with ISR.

Model validation showed an ROC AUC of 0.752 (95% confidence interval 0.702–0.803, Figure 5), indicating good discrimination. After 1,000 bootstrap internal validation replicates, the mean AUC was 0.758 (95% confidence interval: 0.709–0.807). The histogram of AUC values (Figure 6) approximates a normal distribution (peak at approximately 0.75), with 94.6% of values between 0.70 and 0.80, confirming good model stability. The Hosmer–Lemeshow goodness-of-fit test showed no significant difference between expected and observed probabilities (*P* = 0.320); the calibration curve is shown in Figure 7.

**Figure 8:**
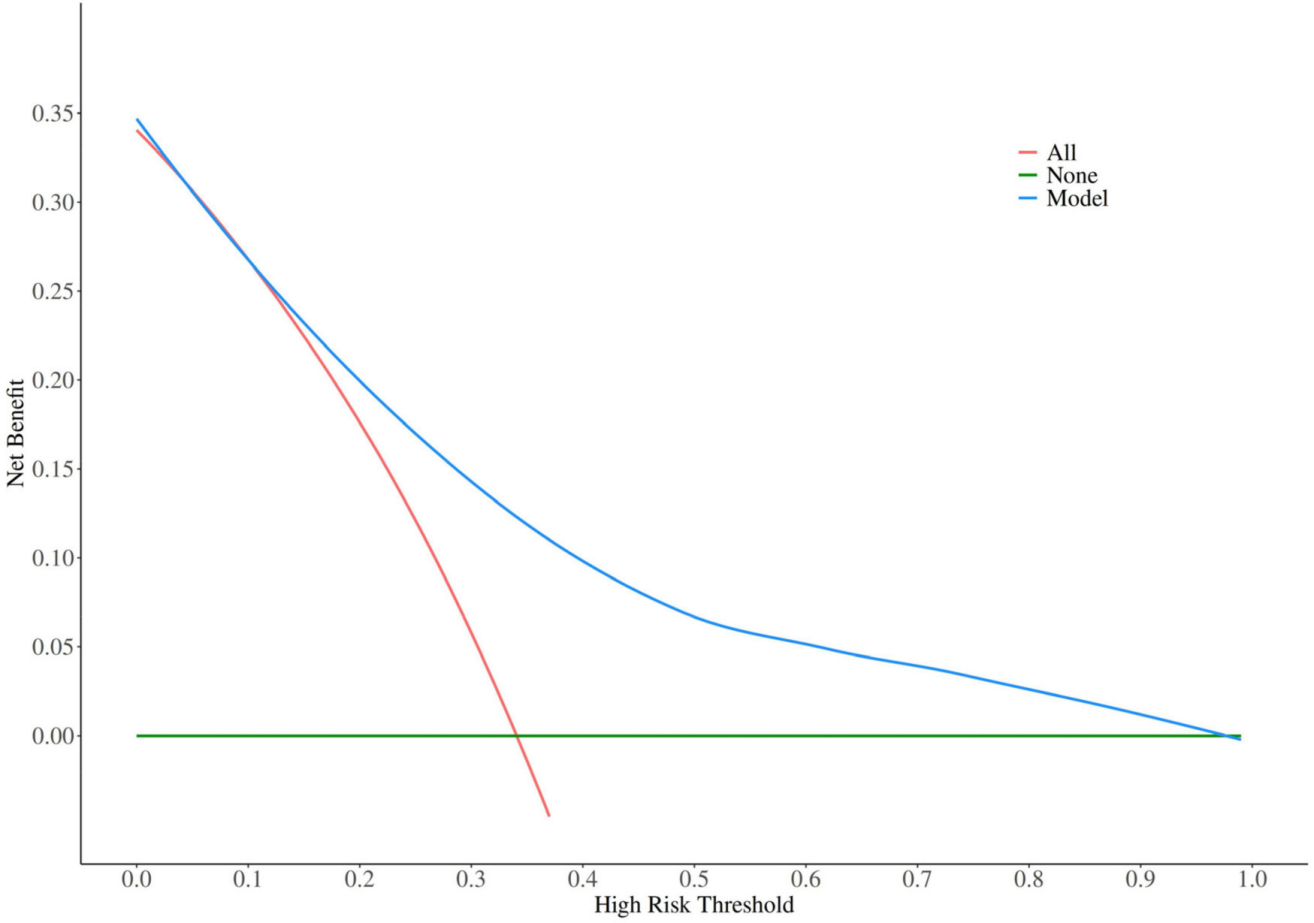
Decision curve analysis for the ISR prediction nomogram

Decision curve analysis (Figure 8) indicated high predictive accuracy and clinical utility across a wide threshold probability range. The DCA demonstrated that the nomogram provided superior net clinical benefit compared with the “treat-all” or “treat-none” strategies across a wide range of threshold probabilities (approximately 10%–60%). In the present clinical context, net benefit is defined as the difference between the proportion of true positives and the weighted proportion of false positives at a given threshold probability, where the weighting factor [threshold probability/(1 − threshold probability)] reflects the relative clinical harm of unnecessary intervention (e.g., bleeding risk) vs. the benefit of preventing ISR.

## 4 Discussion

PCI is the primary method of revascularization for patients with coronary heart disease. ISR remains a major issue affecting the long-term prognosis of patients after a PCI. This study specifically addresses a limitation of previous models, which predominantly focused on preprocedural factors and ignored the importance of postprocedural antiplatelet therapy. Recognizing the high prevalence of CYP2C19*2/*3 alleles in East Asian populations and its impact on clopidogrel metabolism, we incorporated pharmacogenetic testing to strictly verify the antiplatelet hyporesponsiveness measured by TEG. This integrated approach ensures that the predictors identified—specifically the low ADP and AA inhibition rates—represent stable biological responses, thereby enhancing the scientific rigor of the variable definition process within this population. Clinical information was collected using a defined postoperative follow-up cycle and stringent inclusion/exclusion criteria. LASSO regression was then applied to high-dimensional variables to eliminate collinear factors and precisely screen for core predictors. This ensured variable independence while simplifying the model. The ultimately established nomogram model was validated using DCA, demonstrating a wide threshold probability range. This indicates its utility as an effective tool for predicting ISR after a PCI and confirms its strong clinical applicability. Furthermore, this study addresses a gap in previous research by introducing dynamic coagulation monitoring during the post-PCI antiplatelet treatment phase.

### 4.1 Key findings in model construction

This study found that patients in the ISR group were significantly older than those in the non-ISR group (64.83 ± 10.88 vs. 61.83 ± 10.23 years, *P* = 0.007). Significant differences in antiplatelet drug reactivity were observed: Patients with ISR had higher proportions of decreased ADP inhibition (71.53% vs. 43.35%, *P* < 0.001) and decreased AA inhibition (30.15% vs. 6.08%, *P* < 0.001). In addition, NT-proBNP levels were significantly higher in the ISR group (120.00 vs. 54.83 pg/mL, *P* < 0.001). A coagulation analysis revealed a shortened TEG *R*-value (5.39 ± 1.19 vs. 5.82 ± 1.11 min, *P* < 0.001), increased CI (1.30 vs. 0.20, *P* < 0.001), elevated FIB (3.01 vs. 2.81 g/L, *P* = 0.005), and higher D-dimer levels (0.34 vs. 0.25 μg/mL, *P* < 0.001) in the ISR group, indicating a more pronounced hypercoagulable state. These results suggest that advanced age, antiplatelet drug resistance, and a hypercoagulable state are significant risk factors for ISR, while cardiac dysfunction may exacerbate vascular injury through hemodynamic changes, further promoting ISR. These predictors provide important evidence for clinically identifying high-risk ISR patients.

### 4.2 Analysis of key risk factors

#### 4.2.1 Insufficient platelet inhibition promotes ISR

Platelet reactivity is a key determinant of patient outcomes, with ADP and AA inhibition rates serving as measures of this reactivity (23). High platelet reactivity after a stent implantation correlates strongly with clinical prognosis; insufficient ADP/AA inhibition is a hallmark of antiplatelet drug resistance (24), leading to sustained platelet activation. Persistent platelet activation promotes inflammatory cytokine release and accelerates smooth muscle cell migration and proliferation (25, 26). Inflammation triggers early vascular injury, driving neointimal hyperplasia and luminal damage (27). Stent implantation, acting as a foreign body, induces immune responses and damages the arterial wall, exacerbating local inflammation (28, 29). Subsequently, activated platelets, fibrinogen, neutrophils, monocytes, and lymphocytes form an inflammatory cycle sustaining the process (30). Driven by stent-associated inflammation, ISR progression features increased neutrophils, platelets, and monocytes and decreased lymphocytes. Elevated platelet counts indicate a prothrombotic and proinflammatory state, while lymphopenia suggests uncontrolled inflammation (31, 32). Platelet-neutrophil interactions recruit monocytes, promote inflammatory cytokine release, maintain inflammation, and ultimately drive neointimal hyperplasia, atherosclerosis progression, thrombosis, and ISR (27, 33, 34). Therefore, monitoring and managing antiplatelet drug resistance is crucial for improving long-term outcomes after a stent implantation.

#### 4.2.2 Advanced age and impaired vascular endothelial repair

Advanced age is closely associated with diminished vascular endothelial repair capacity and a proinflammatory state, potentially accelerating neointimal hyperplasia (35). Aging involves endothelial cell (EC) senescence and vascular dysfunction, contributing to increased cardiovascular risk. Pathophysiological mechanisms include dysregulation of vascular tone, increased endothelial permeability, arterial stiffening, impaired angiogenesis and repair, and reduced mitochondrial biogenesis in ECs (36). Increased production of reactive oxygen and nitrogen species (RONS) with age consumes nitric oxide (NO)—essential for endothelial vasodilation and inhibiting platelet/leukocyte adhesion (37)—and accelerates LDL oxidation. Oxidized LDL deposits in the vessel wall, further damaging endothelial integrity and hindering self-repair (38). Chronic inflammation in aging elevates cytokines (IL-1β, IL-6, TNF-α), which downregulate endothelial nitric oxide synthase (eNOS) expression, reducing NO production (39), and promote cell adhesion molecule (CAM) expression, increasing leukocyte adhesion to the endothelium and exacerbating injury (40, 41). Consequently, impaired repair mechanisms fail to adequately respond to persistent injury, favoring pathological repair processes like neointimal hyperplasia (42), forming a critical pathological basis for age-related vascular aging and atherosclerosis (43, 44). Studies suggest that rejuvenation strategies [e.g., bone marrow rejuvenation improving endothelial progenitor cell (EPC) function] can accelerate re-endothelialization and reduce neointima in aged mice (45). Inhibiting inflammatory signaling and lifestyle interventions like regular exercise (activating AMPK, reducing oxidative stress, increasing NO bioavailability) may also mitigate age-related vascular dysfunction (46).

#### 4.2.3 Thromboelastography for monitoring postprocedural hypercoagulability

TEG dynamically assesses global coagulation function (47). The parameters are *R*-value, coagulation time (*K*-value), α-angle, MA, Ly30, EPL, and CI. A shortened *R*-value indicates accelerated coagulation initiation, suggestive of hypercoagulability and increased thrombotic risk. It is noteworthy that in the present study, the values for EPL and Ly30 tended toward 0.000 (0.000–0.000) in both the ISR and non-ISR groups. From a biological perspective, this accurately reflects the fibrinolytic system characteristics of patients with CAD in the stable phase 12–14 months post-PCI. Unlike patients in the acute phase or those undergoing major surgical procedures, patients with stable CAD receiving long-term statin and DAPT therapy benefit from a degree of protected vascular endothelial function, which typically results in fibrinolytic activity remaining at extremely low basal levels. Furthermore, the detection window of conventional TEG assays has a technical threshold that may not capture trace variations in fibrinolysis. Therefore, in this specific cohort, these fibrinolytic indicators functioned primarily to exclude the risk of systemic hyperfibrinolysis rather than serving as primary predictive factors for ISR. Elevated CI reflects enhanced clot strength and stability, also associated with thrombotic propensity (48). The utility of TEG extends beyond PCI to trauma and other surgeries. For instance, TEG detects procoagulant imbalance and hyperfibrinolysis in severe burns, which correlate with mortality (49). An analysis of intraoperative TEG in 124 liver transplant recipients showed a shortened *R*-time despite INR > 2 in 37.7% of cases; four out of six patients with early hepatic artery thrombosis had a shortened *R*-time (50), suggesting that TEG identifies hypercoagulability missed by conventional tests. Similarly, TEG-defined hypercoagulability (*R* < 5 min) predicted higher rates of symptomatic intracranial hemorrhage (ICH) and early neurological deterioration (END) in patients with acute ischemic stroke compared with those with *R* ≥ 5 min (51). Therefore, TEG parameter monitoring holds significant value for peri- and postprocedural management in various settings.

#### 4.2.4 Mechanistic role of NT-proBNP in ISR pathogenesis

Although NT-proBNP did not reach statistical significance in the multivariable logistic regression analysis, its inclusion in the nomogram as a key predictor underscores its substantial incremental value in risk stratification. Mechanistically, elevated NT-proBNP levels reflect increased ventricular wall stress and subclinical cardiac dysfunction, even in patients with preserved ejection fraction. This neurohormonal activation fosters a proinflammatory and profibrotic microenvironment that impairs endothelial healing following stent-induced vascular injury. Specifically, heightened wall tension increases shear stress at the stent edges, promoting smooth muscle cell migration and extracellular matrix deposition, which ultimately drives neointimal hyperplasia. Furthermore, the hemodynamic alterations associated with elevated NT-proBNP exacerbate a prothrombotic state—consistent with the synchronized elevation of fibrinogen and D-dimer observed in the ISR group of this study—thereby delaying re-endothelialization and amplifying local inflammatory responses. These pathways align with recent evidence suggesting that perioperative NT-proBNP elevation is associated with an accelerated onset of severe ISR and an increased risk of major adverse cardiac events, indicating that NT-proBNP serves not merely as a biomarker of heart failure but as an active participant in adverse postoperative vascular remodeling. This model integrates predictors covering the core mechanisms of ISR (coagulation–inflammation–endothelial dysfunction) (52, 53), while emphasizing the clinical value of antiplatelet therapy monitoring.

### 4.3 Model positioning analysis

#### 4.3.1 Positioning and clinical significance of the predictive model

Compared with existing predictive models, the model developed in this study exhibits distinct advantages in variable selection and clinical positioning. Previous inflammation-related ISR nomograms, such as those developed by Luo et al. based on monocyte counts, Gensini scores, and the neutrophil-to-lymphocyte ratio (NLR) (21), or the machine learning model by Hou et al. utilizing the aggregate index of systemic inflammation (AISI), achieved high discriminative power with AUC values of 0.847 and 0.9569 (54), respectively. However, these models predominantly focused on preprocedural inflammatory and anatomical indicators, which are largely fixed and non-modifiable, thus offering limited guidance for postprocedural therapeutic adjustments.

In contrast, our study innovatively incorporates dynamic TEG monitoring data to evaluate antiplatelet efficacy, shifting the focus toward modifiable post-PCI factors. To mitigate the risk of false positives inherent in relying solely on TEG inhibition rates, we employed pharmacogenetic testing for secondary verification of hyporesponsiveness, enabling more precise identification of true drug resistance. Given the substantial interindividual variability in antiplatelet response, utilizing TEG to monitor low responders facilitates the optimization of treatment regimens, reduces ischemic events, and minimizes bleeding risks. Consequently, this model provides a novel, practical tool for ISR risk assessment that bridges the clinical gap between postprocedural monitoring and individualized management.

#### 4.3.2 DAPT duration and population-specific considerations

With regard to inclusion criteria, our study required a minimum of 12 months of DAPT, reflecting the clinical landscape of East Asia during the study period (2018–2024). In this population, the high prevalence of CYP2C19 loss-of-function alleles correlates with elevated ischemic risk (55), justifying the prolonged DAPT strategy consistent with guidelines for patients without high bleeding risk. While contemporary ESC/ACC guidelines increasingly favor shortened, individualized DAPT to reduce bleeding exposure, our model was specifically designed to support such precision decision-making. By providing early risk stratification during stable DAPT (≥6 months), the nomogram assists clinicians in identifying candidates for de-escalation, intensification, or extension of therapy.

#### 4.3.3 Clinical utility and DCA

DCA demonstrated a significant clinical net benefit for our model within a threshold probability range of 10%–60%. Importantly, since DCA is inherently influenced by the prevalence of the outcome, it should be noted that our findings reflect an ISR prevalence rate of 34.2% within this cohort. Therefore, the generalizability of the model's clinical net benefit to populations with significantly different ISR rates warrants further external validation. In this context, “net benefit” represents the proportion of true positives (correctly identified high-risk ISR patients) minus the weighted proportion of false positives (unnecessary interventions) (56). Clinicians can utilize the nomogram during the 6–12-month follow-up to calculate individual ISR probabilities. For patients with a predicted probability exceeding 30%–35% (a threshold adjustable based on local bleeding risk profiles), the following evidence-based interventions are recommended:

1. Switching P2Y12 inhibitors: transitioning from clopidogrel to more potent agents (e.g., ticagrelor 90 mg BID or prasugrel 10 mg QD) to overcome documented hyporesponsiveness.
2. DAPT extension: extending the duration of therapy beyond 12 months in low-bleeding-risk patients.
3. Enhanced surveillance: scheduling earlier or more frequent angiographic or non-invasive follow-ups.
4. Risk factor management: intensifying control of modifiable factors such as hypertension, dyslipidemia, and diabetes.

Conversely, low-risk patients (probability <20%) may safely continue or undergo de-escalation of standard DAPT. This threshold-guided strategy translates the model's statistical benefit into an actionable clinical pathway.

Our framework integrates with previous evidence from large-scale trials (e.g., GRAVITAS, ARCTIC, and TAILOR-PCI) that investigated platelet function or genotype-guided therapy (57). While those trials often showed that routine, serial monitoring to guide universal dose intensification failed to improve overall outcomes, our model does not propose such a repetitive monitoring mandate. Instead, it serves as a milestone-based risk stratification tool. Although the model requires TEG measurements as an input, we advocate for its use as a targeted assessment at a stable postoperative time point (6–12 months). In populations with high pretest probabilities of drug resistance, such as the East Asian cohort in this study, this one-time assessment facilitates the identification of high-risk individuals for selective optimization, bridging the gap between universal treatment and precision cardiovascular care.

We acknowledge that the requirement for a planned 12–14-month angiographic follow-up may introduce a “survivor bias” by excluding patients with early major events. However, the core value of the model lies in identifying high-risk individuals during the stable DAPT phase to prevent such adverse outcomes through early intervention. Furthermore, procedure-related variables [e.g., stent diameter, length, residual stenosis, and lesion complexity (58)] were not captured in a standardized manner in this retrospective dataset and were thus excluded. While these are established predictors, our model deliberately prioritizes modifiable, dynamically monitorable postprocedural indicators—specifically TEG-derived antiplatelet efficacy—to maximize clinical operability.

Finally, this cohort was focused on patients from Southern Fujian, China, where the high prevalence of CYP2C19 variants makes the model particularly relevant to East Asian populations at high ischemic risk. While this population specificity enhances local utility, it may limit direct generalizability to Western populations with different genetic profiles. Future multicenter, multiethnic prospective studies are warranted to validate the stability of this model across diverse populations and to explore the integration of optical coherence tomography (OCT) imaging to further refine ISR prevention strategies.

## 5 Conclusions

In summary, this study constructed and validated a prediction model for ISR after a PCI in a population from Southern Fujian Province, China. Based on thromboelastography parameters (ADP inhibition rate, AA inhibition rate, *R*-value), age, and NT-proBNP, the model was presented as a nomogram. Evaluation through ROC curve analysis, Hosmer–Lemeshow test, decision curve analysis, and bootstrap internal validation confirmed its good discrimination, calibration, clinical utility, and stability. This model provides a basis for decision-making regarding individualized antiplatelet therapy.

## 6 Limitations

This study has several limitations. The retrospective, single-center design and the exclusion of 36.5% of eligible patients due to missing laboratory data may introduce selection bias. Procedural factors like stent length and lesion complexity were not included due to a lack of standardized recording; however, the model focuses on modifiable postprocedural TEG parameters to maximize clinical utility in antiplatelet adjustment. Genetic differences, specifically the high frequency of CYP2C19 variants in East Asians, may also limit the model's external validity in Western cohorts. Moreover, the reliance on scheduled angiography introduces survivor bias, as patients with early adverse events or those declining invasive follow-up were not captured.

Despite these constraints, our nomogram serves as a robust evidence-based tool for postoperative ISR risk stratification. By enabling early identification of high-risk candidates for DAPT intensification and protecting low-risk patients from unnecessary bleeding exposure, this model supports the transition toward more precise and individualized cardiovascular care. Future research incorporating multicenter data and advanced imaging like OCT will be essential to further enhance its predictive performance.

Last, the clinical utility demonstrated by DCA is specific to the ISR prevalence of our study, which may limit the model's direct applicability in clinical settings where the baseline risk of restenosis differs significantly.

## Data Availability

The original contributions presented in the study are included in the article/Supplementary Material; further inquiries can be directed to the corresponding author.
